# A Method for Detecting and Analyzing Facial Features of People with Drug Use Disorders

**DOI:** 10.3390/diagnostics11091562

**Published:** 2021-08-28

**Authors:** Yongjie Li, Xiangyu Yan, Bo Zhang, Zekun Wang, Hexuan Su, Zhongwei Jia

**Affiliations:** 1 School of Public Health, Peking University, Beijing 100191, China; li.yongjie@outlook.com (Y.L.); yanxiangyu@bjmu.edu.cn (X.Y.); bibibabo@pku.edu.cn (B.Z.); wangzekun@bjmu.edu.cn (Z.W.); suhexuan@bjmu.edu.cn (H.S.); 2 Medical Informatics Center, Peking University, Beijing 100191, China; 3 Center for Intelligent Public Health, Institute for Artificial Intelligence, Peking University, Beijing 100191, China; 4 Center for Drug Abuse Control and Prevention, National Institute of Health Data Science, Peking University, Beijing 100191, China

**Keywords:** drug use disorders, machine learning, clinical screening, feature recognition, deep learning, image visualization

## Abstract

Drug use disorders caused by illicit drug use are significant contributors to the global burden of disease, and it is vital to conduct early detection of people with drug use disorders (PDUD). However, the primary care clinics and emergency departments lack simple and effective tools for screening PDUD. This study proposes a novel method to detect PDUD using facial images. Various experiments are designed to obtain the convolutional neural network (CNN) model by transfer learning based on a large-scale dataset (9870 images from PDUD and 19,567 images from GP (the general population)). Our results show that the model achieved 84.68%, 87.93%, and 83.01% in accuracy, sensitivity, and specificity in the dataset, respectively. To verify its effectiveness, the model is evaluated on external datasets based on real scenarios, and we found it still achieved high performance (accuracy > 83.69%, specificity > 90.10%, sensitivity > 80.00%). Our results also show differences between PDUD and GP in different facial areas. Compared with GP, the facial features of PDUD were mainly concentrated in the left cheek, right cheek, and nose areas (*p* < 0.001), which also reveals the potential relationship between mechanisms of drugs action and changes in facial tissues. This is the first study to apply the CNN model to screen PDUD in clinical practice and is also the first attempt to quantitatively analyze the facial features of PDUD. This model could be quickly integrated into the existing clinical workflow and medical care to provide capabilities.

## 1. Introduction

A drug use disorder, including drug abuse and drug dependence, is the persistent use of drugs despite substantial mental, physical, or behavioral harm. These disorders lead to adverse consequences, more commonly caused by illicit drugs (including stimulants, depressants, and hallucinogens), physiological withdrawal symptoms, and the inability to reduce or stop consuming drugs [1]. Drug use disorders caused by illicit drug use are significant contributors to the global burden of disease, and directly led to 20 million disability-adjusted life-years (DALYs) in 2010—accounting for 0.8% of global all-cause DALYs [2]. The Global Burden of Disease Study showed that 35 million suffered from drug use disorders and required treatment services, and 750,000 people died as a result of illicit drug use in 2017 [3]. Therefore, it is vital to recognize the early signs of drug use disorders, and provide early intervention before addiction takes hold, which is essential to ensure the most robust chances of successful recovery.

With the increase in patients using illicit drugs, primary care clinics and emergency departments (EDs) are facing challenges. Less than 20% of primary care physicians claimed to have sufficient expertise to identify illegal drug use and provide treatment suggestions for patients with drug use disorders [4]. ED physicians usually make diagnosis and treatment decisions based on the patient’s self-reported substance use. However, studies have shown that patients tend to deny or underreport illicit drug use [5]. Routine illicit drug testing methods were adopted by clinicians to assess patients’ addictions and perform further treatment [6]. However, within a short time frame, these tools were easily tampered with, or results were faked [7]. In busy primary care practices, the high time cost of screening prevented it from being integrated into the clinical workflow [8]. Due to the disadvantages of traditional illicit drug testing, it is necessary to try new detection methods.

People with drug use disorders (PDUD) can be recognized and diagnosed by physicians based on the presence of multiple physical, psychological, emotional, behavioral symptoms and signs in clinical practice. Among them, physical signs could be an important and feasible target for detecting PDUD in clinical screening. Related research indicated that people with severe drug use disorders might have apparent physical signs, especially facial features, such as flush cheeks or redness around the mouth and nose, facial acne, and sudden weight loss, which could be easily detected [9]. However, most PDUD lack these strong indicators, and in early clinical screening, they are more likely to be ignored. Therefore, for better practice in clinical screening, facial feature detection technology for PDUD using convolutional neural networks (CNN) is meaningful. CNN has an advantage over other machine learning algorithms in feature learning and has made breakthroughs in computer vision. It can automatically extract features based on an end-to-end model without manually transforming features. The aim of this study was to detect and classify facial images of PDUD and the general population (GP) by CNN to assist clinical screening.

### Related Works

Although some research has been completed on the detection of PDUD using deep learning, the relative research on drug use has provided new ideas. Snorting illicit drugs could cause permanent damage to a person’s nose [10]. Some illegal drugs, such as cocaine, act as powerful stimulants that suppress appetite and lead to undernourishment for a long period of time [11]. Rapid weight loss could cause the body to begin consuming muscle tissue and facial fat, accelerating biological aging, and leading to face distortion [12,13]. Thus, abnormalities in some/whole face areas might also be indicators of PDUD. A previous study has pointed out that a significant increase in facial asymmetry in methamphetamine abusers [14].

Deep learning has been used actively in medical imaging, such as disease detection, medical image segmentation. As traditional methods reach their performance limits on images, CNN have started to dominate because of their good results on varying image classification tasks [15]. Shankar [16] proposed a deep learning algorithm based on the assessment of color fundus photographs to predict diabetic retinopathy (DR) progression in patients, and the clinical trial showed its potential in early identification of patients at the highest risk of DR, allowing timely referral to retina specialists and initiation of treatment. William [17] trained a CNN model to improve breast cancer detection on screening mammography, and it achieved an area under the curve (AUC) of 0.927 in the training dataset. The model could accurately locate the clinically significant lesions and base predictions on the corresponding portions of the mammograms. It was also effective in reducing false positives and false negatives in clinical screening. In the end-to-end training method, CNN models can directly convert input data into an output prediction without constructing complicated hand-craft features, and the parameters of the intermediate layers are automatically learned, and feature extraction is done during the training process. In this paper, a large-scale image dataset was prepared, and a CNN model with higher accuracy for screening patients with drug use disorders was proposed, making it promising for clinical applications.

## 2. Materials and Methods

### 2.1. Study Design and Procedure

**Figure 1:**
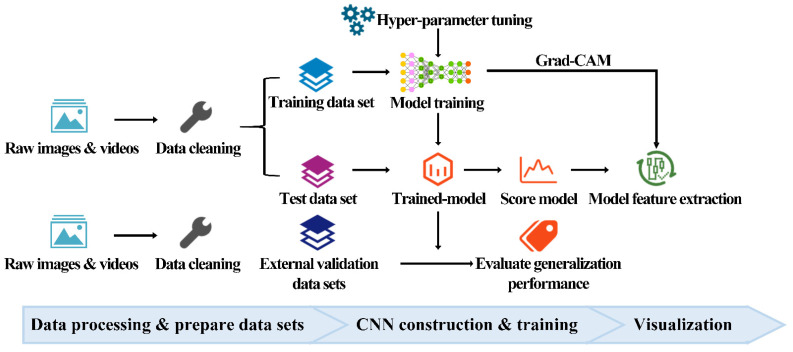
The entire flowchart of this study.

**Figure 2:**
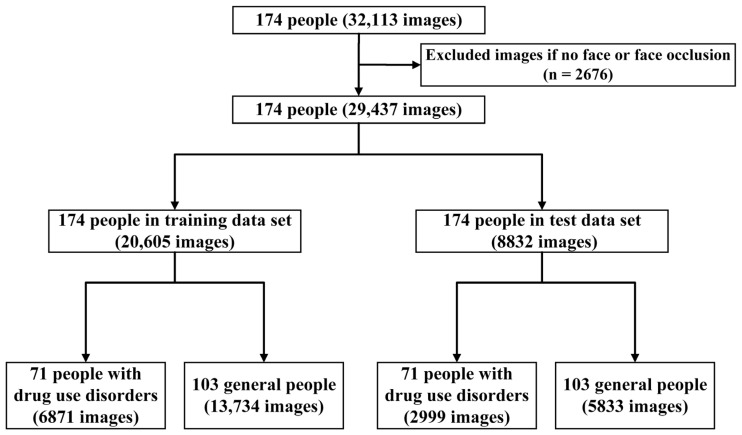
The flow of data source and datasets collection.

**Figure A1:**
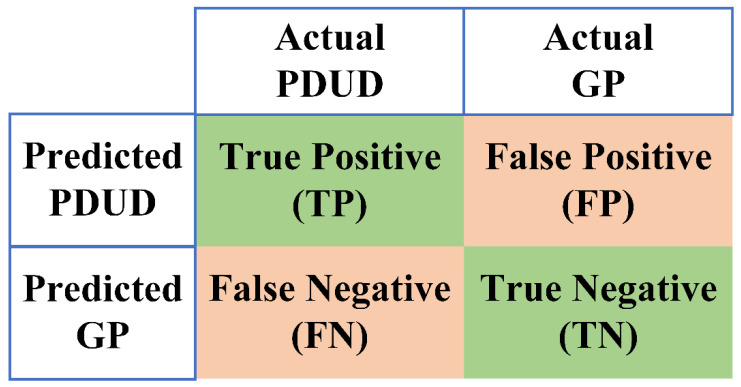
The definitions of true positive, false positive, false negative, and true negative.

Our study consisted of three main processes, as shown in Figure 1. First, 2416 images and 256 videos of 71 PDUD, and 103 videos of 103 GP were collected. PDUD were collected from a mobile health (mHealth) app (detailed information of the app can be found in Appendix A). The time range of the data in the mHealth app was from 30 October 2017 to 31 January 2020. The videos of GP were collected from the Internet. Video data captured a frame every 3 s and was saved as an image. Second, the images of PDUD (10,447) and the GP (21,666) in the dataset were preprocessed to obtain a clear facial image, and invalid or blurred ones would be removed. Third, to eliminate external distracting information, such as the background, clothes, or accessories, face cropping was performed on them to remove the images of face occlusion. To facilitate the batch processing of the CNN model, all images were resized to 224 × 224 pixels. After the above preprocessing, the images of PDUD (9870) and the GP (19,567) were merged (Figure 2). Based on the 70/30 principle, these images were shuffled and randomly divided these images into a training dataset and a test dataset. The CNN model was trained in the training dataset and calculated the accuracy, sensitivity, and specificity in the test dataset (Appendix B Figure A1).

**Figure 3:**
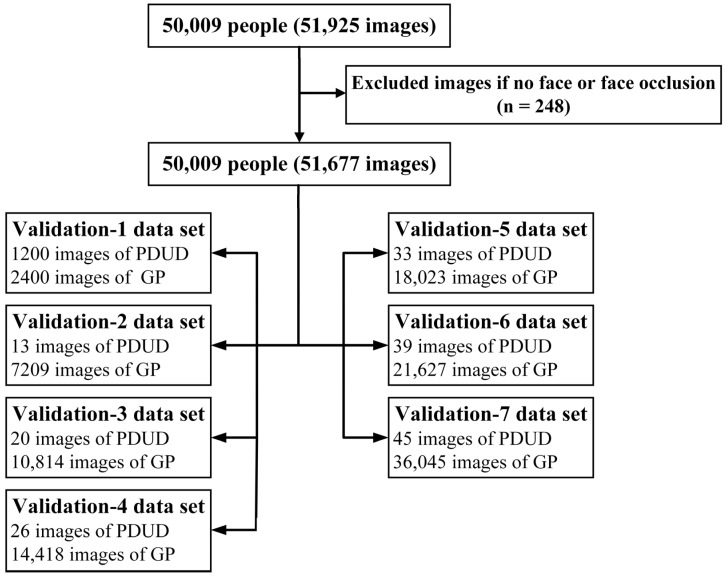
The flow of external validation datasets collection and data distribution in each validation dataset.

Before considering using the model for clinical prediction, it is essential that its performance be empirically evaluated in datasets that were not used to develop the model [18]. Therefore, external validation datasets were prepared to evaluate the trained model. Another nine videos of nine PDUD and 50,000 images of 50,000 GP were collected, among which the PDUD data were provided by the local administrative department of Jinan City, Shandong Province, China, and the GP data were collected from the public database [19]. The videos of PDUD also underwent a similar video processing flow. After the above video processing, the images of PDUD and GP were 1925 and 50,000, respectively. Those images were filtered out, which were unclear or blurred images, and when preprocessing was complete, there were 1677 images of PDUD and 50,000 images of GP, respectively. The external validation datasets included validation 1 dataset and validation 2 to validation 7 datasets. For the validation 1 dataset, the data distribution was consistent with the training/test dataset, which was used to evaluate the performance of the trained model in the face of new data. In addition, considering the prevalence of drug use disorders in the clinic, the number of images in the validation 2 dataset was calculated as the required minimum sample number based on the prevalence in China (1.80%, power = 0.90, α = 0.05) [20]. Moreover, to further evaluate the performance of our trained model under the real-world scenario, they expanded the 1.5, 2, 2.5, 3, and 5 times based on the sample’s number in the validation 2 dataset, and obtained the validation 3–7 datasets, respectively (Figure 3). With reference to the number of images required in each validation dataset, they were randomly selected from these images (51,677) of PDUD and GP. Finally, the performance of the trained model was evaluated on these seven validation datasets. The above preprocessing of images was completed by the Dlib library, and the sample size was calculated by PASS version 11.0 [21,22,23,24].

### 2.2. CNN Construction and Training

CNN models were trained in the training dataset and tested in the test dataset to extract valid facial information from a large sample of images. Since the labels of the dataset had binary labels, the task was designed as a binary classification.

To find the appropriate model architecture, we analyzed the mainstream CNN models with transfer learning: Vgg-19, Inception, and Resnet-18 [25,26,27]. Then, the attention technique and the pre-trained model were introduced for training to improve the accuracy of the CNN model. The attention technique was used to make our CNN learn and focus more on the important information of images [28]. A pre-trained model was a saved network that was previously trained on a large dataset, typically on a large image dataset similar to the training target [29]. Therefore, a pre-trained Resnet-18 model in MS-Celeb-1M was chosen, which was a database for large-scale face recognition [30]. In addition, we tried different freezing layers configurations to compare the performance of models during transfer learning. The configurations include: (1) Training a CNN from scratch; (2) freezing all the layers but training the last fully connected layer; and (3) freezing all the layers but training the last five ones. Next, the training of the CNN model involved multiple hyperparameters, and the performance of the CNN models on the test dataset could be improved by adjusting different parameters. To obtain better parameters, different experiences were designed to adjust various parameters, while avoiding over-fitting and under-fitting problems on the dataset (Table 1). The adjusted strategy included: (1) Different learning rates (LR); (2) whether to use batch normalization (BN); (3) whether to use a pre-trained model; (4) whether to initialize the weights in the layers of the models. Moreover, the optimization algorithms stochastic gradient descent (SGD) and adaptive moment estimation (Adam) were applied to select better training algorithms, respectively [31] (Detailed training information can be found in Appendix B and Appendix C).

When the loss of the models on the training dataset no longer decreased, the training ended. By comparing the accuracy of models with different parameters on the test dataset, the model with the best accuracy was chosen as the final CNN model. The sensitivity and specificity of the test dataset were calculated to evaluate the model comprehensively. In the external validation datasets, the best-performance model was used to calculate the accuracy, sensitivity, and specificity of the seven external validation datasets. The entire code of image analysis was done with open-source Python 3.6, and the construction of the CNN networks was implemented based on the PyTorch 1.3 [32] (Appendix B Algorithm A1). All networks were trained on an NVIDIA GeForce GTX 2080Ti.

### 2.3. Quantitative Analysis of Facial Features and Visualization

**Figure 4:**
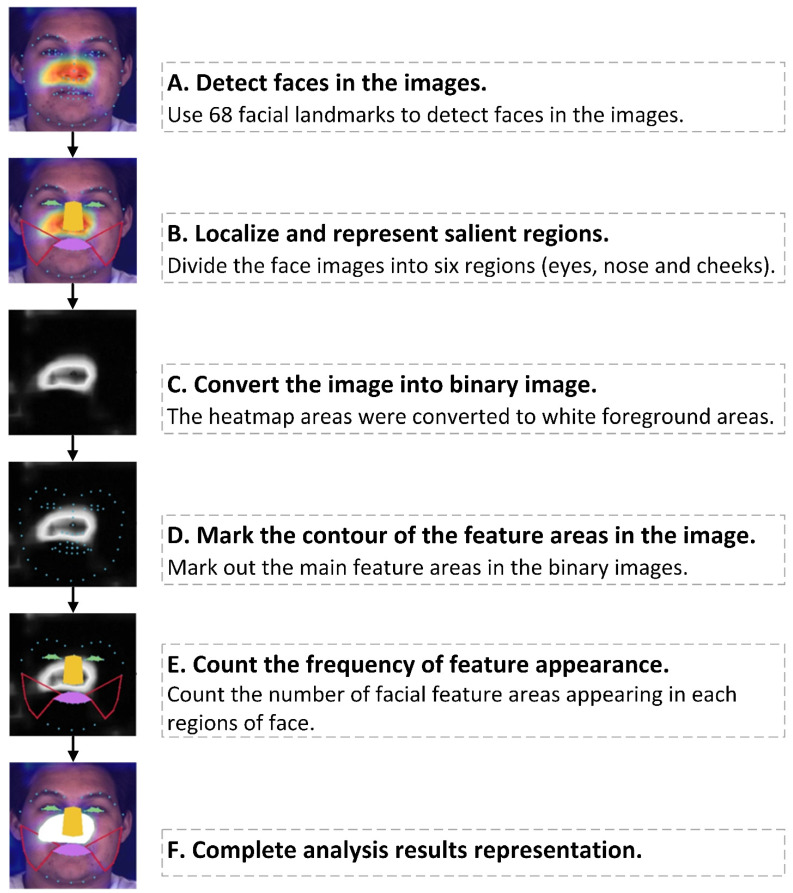
The quantitative facial feature analysis process. Note: The picture is for demonstration purposes only. The person in the picture has no connection with any use of illicit drugs. The data are from the BP4D dataset [35,36].

**Figure A2:**
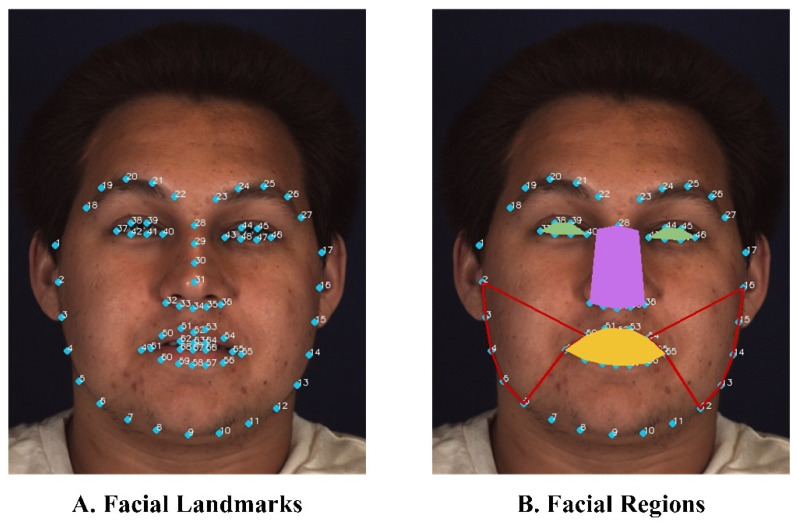
Diagram of facial regions division. (**A**) The 68 coordinates to determine the facial structure. (**B**) The different colored lines represent different facial regions. Green is the eye area, red is the cheek, yellow is the mouth, and purple is the nose. Note. The picture is for demonstration purposes only. The data are from the BP4D dataset [35,36].

The interpretability of the CNN model is useful to explain why it predicts what it predicts. The feature map referred to the result of output captured by the filter on the output of the previous layer of the network. The gradient weighted class activation mapping (Grad-CAM) technique was applied to visualize the high-dimension information of the CNN model [33]. Then we quantitatively analyzed whether there were significant differences in features between PDUD and the GP in each facial area. The analysis process that automatically counted the number of facial features in different facial areas in the input images was constructed. The complete analysis process was divided into the following six steps (Figure 4). First, a facial mark detector was applied to produce 68 coordinates that were mapped to the structure of the face, and then the entire face was divided into six different areas: The left and right eyes, the nose, the left and right cheeks, and the mouth (Figure 4B and Appendix B Figure A2). The image with heatmap as the input data was converted into a binary image. In the binary image, the heatmap area tended to be white, and the other positions tended to be the opposite black. (Figure 4C). In the fourth step, the Gaussian Blur operation was performed on the binary images with a Gaussian kernel size of 3 × 3, and then the threshold operation was performed on the binary images. Finally, the contours in the binary images were marked, which were the facial features (Figure 4D). The contours those length or width were less than 10 pixels would be discarded because they were too small to be valid facial features. In the fifth step, the number of times each facial feature appeared in the six facial areas was counted, and the proportion of different areas were calculated (Figure 4E). The sixth step shows the result of the demonstration (Figure 4F). The above steps were carried out by OpenCV-Python [34]. Finally, the characteristics of PDUD and the GP in different facial areas were compared by the chi-square test using SPSS version 22.0 (IBM Corporation, Armonk, NY, USA).

## 3. Results

### 3.1. CNN Model Training and Performance

After excluding 2676 images because of the lack of clear or complete facial images, the training dataset consisted of 6871 PDUD images and 13,734 GP images, and the test dataset consisted of 2999 PDUD images and 5833 images of the GP (Figure 2).

For the three models, the Resnet-18 achieved a better result, and it was only trained in the last five convolutional layers (Table 2). Compared with Vgg-19 and Inception, there was an increase of 2–23 percentage points in Resnet-18. Therefore, Resnet-18 was the suitable architecture in this study. In addition, the results showed that both attention technique and pre-trained model would help Resnet-18 to achieve better performance, with an increase of about 4 and 10 percentage points, respectively (Appendix B Table A1). Therefore, the pre-trained model had more advantages for transfer learning. Regarding the different optimization algorithms, our results show that SGD was better than Adam in improving the model scores (Appendix B Table A2).

**Figure 5:**
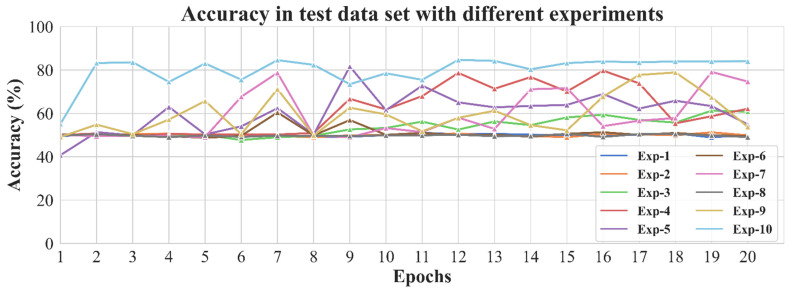
The accuracy of experiments with different parameters on the test dataset. Exp-1 was experiment-1 (Vgg-19, learning rate 0.1, without batch normalization, without pre-trained, without weight initialization). Exp-2 was experiment-2 (Vgg-19, learning rate 0.01, without batch normalization, without pre-trained, without weight initialization). Exp-3 was experiment-3 (Vgg-19, learning rate 0.01, with batch normalization, without pre-trained, without weight initialization). Exp-4 was experiment-4 (Resnet-18, learning rate 0.1, with batch normalization, without pre-trained, without weight initialization). Exp-5 was experiment-5 (Resnet-18, learning rate 0.01, with batch normalization, without pre-trained, without weight initialization). Exp-6 was experiment-6 (Resnet-18, learning rate 0.1, with batch normalization, without pre-trained, with weight initialization). Exp-7 was experiment-7 (Inception, learning rate 0.01, with batch normalization, without pre-trained, without weight initialization). Exp-8 was experiment-8 (Inception, learning rate 0.1, with batch normalization, without pre-trained, without weight initialization). Exp9 was experiment-9 (Resnet-18 with attention, learning rate 0.1, with batch normalization, without pre-trained, without weight initialization). Exp-10 was experiment-10 (Resnet-18, learning rate 0.1, with batch normalization, with pre-trained, without weight initialization).

Then, in the 10 experiments which aimed to adjust the parameters of the CNN model to improve the accuracy of the test dataset, the best accuracy of each experiment was 51.03%, 51.25%, 61.23%, 79.72%, 81.64%, 60.43%, 79.13%, 50.55%, 78.88%, and 84.68% (Table 1, Appendix B Table A1). Finally, the Resnet-18 model with the best accuracy of 84.68% was selected, and the parameters included the learning rate of 0.1, using batch normalization technology, and using the pre-trained model in the MS-Celeb-1M dataset (Figure 5). Moreover, regarding the test dataset, the sensitivity and specificity of the model were 87.93% and 83.01%, respectively (Table 3 and Appendix B Figure A1).

**Figure A4:**
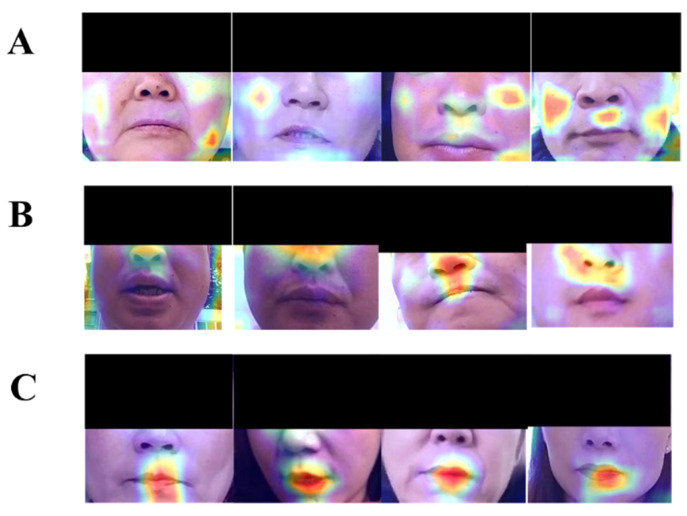
Visualization of facial features of the people with drug use disorders. The heatmap in the figure represents the facial features extracted by the CNN model. (**A**) The heatmap indicates the feature on the cheek area. (**B**) The heatmap indicates the feature on the nose area. (**C**) The heatmap indicates the feature on the mouth area.

According to the sensitivity and specificity of the test dataset, the minimum number of samples was 13 images of PDUD and 7209 images of GP. Then we selected the corresponding amount of data and prepared seven external validation datasets (Figure 3). The performance of the model in seven groups external validation datasets was: The accuracy was higher than 83.69%, the highest was 90.10%, the sensitivity was higher than 80.00%, the highest was 86.67%, and the specificity was higher than 84.25%, the highest was 90.10% (Table 3, Appendix B Figure A1 and Figure A4).

### 3.2. Typical Facial Features of PDUD and Visualization

In the activation heat map on the images, colors highlighted these apparent facial features extracted by the CNN model. In the examples of visualized images of PDUD, rows A, B, and C, respectively, represent the output features in the cheeks, nose, and mouth areas (Appendix B Figure A4). The concentration of facial features recognized by the CNN model was different between PDUD and GP. The proportions of the GP in the six facial feature areas were similar (35.92% in left-eye, 43.31% in right-eye, 40.97% in mouth, 29.02% in the nose, 34.36% in left-cheek, 35.47% of right-cheek). However, the recognizable facial features of drug users were more distinctive and mainly concentrated in the nose (42.98%) areas, left cheek (44.91%), and right cheek (44.85%), and these proportions were much higher than that of the GP (*p* < 0.001) (Table 4).

## 4. Discussions

This study developed and validated an image-based CNN model for screening PDUD. As the most popular CNN architecture in computer vision, the Resnet network showed higher performance with the simple, but effective residual block. Freezing the last five layers also benefited transfer learning and reduced computation time. In addition, the attention technique and pre-trained model in transfer learning were introduced in experiments. Overall, the pre-trained model contributed more to the final scores than the attention module. Considering that the attention mechanism module still needed iterative training to extract the related feature information, but the pre-trained model already contained rich facial information, which also enabled the model to quickly extract facial feature cues of PDUD. Therefore, the Resnet-18 with a pre-trained model was selected as the transfer learning scheme. Based on this, the model achieved a high accuracy of 84.68% on the test dataset by fine-tuning parameters.

The external validation datasets were inspired by the authors of [37], and thus, based on real scenarios. These scenarios were built to evaluate the performance of the model, and the prevalence of PDUD in them was consistent with that in the real scenario. The results showed that our CNN model still maintained a better score, which meant that this model was promising in practical clinical screening. The rapid screening efficiency, simple operation process, and low medical cost enable our model to be quickly integrated into the existing clinical workflow and medical care. The related study found that most primary care physicians were not yet ready to prepare for drug abuse [38]. Our method can be applied to primary care clinics to provide screening services for patients, especially when patients first visit the clinic. The screening can be done prior to the medical encounter or in the waiting room. In addition, our model can also be flexibly deployed on mobile apps. The screening can be done through the patient portal while the patient is at home, and the results can be integrated into the electronic health record to assist primary care physicians in providing appropriate preventive care. This not only alleviates the discomfort of patients during face-to-face screening, but also protects individual privacy. This electronic screening was also supported by patients [8]. On the other hand, our model can be embedded in the admission system of ED to provide the capability to detect drug use disorders quickly. In the routine emergency treatment process, the newly acquired screening capability can help doctors know the patient’s drug use condition and determine further intervention or referral to drug use treatment.

The visualization of the feature maps of our CNN model showed that drug use affected the face of the patients, which was consistent with previous case report studies [39]. The statistical results showed that the significance of PDUD in the nose and cheek areas revealed the potential relationship between drug use patterns and mechanisms of action of drugs and changes in facial tissues, which is the first quantitative analysis of facial features in the related studies of PDUD. On the one hand, the characteristics of the nose area suggested that this may be related to specific drug use patterns. Snorting, sniffing (intranasal delivery), or smoking drugs, is a drug use pattern often chosen by drug users to avoid injection use. Because the mucosa inside the nose is easily accessible, the drugs are quickly absorbed in the form of powder, liquid, or aerosol, which can irritate or infect the nasal tissue [40]. Frequent snorting, sniffing, or smoking illicit drugs will cause a lack of oxygen and nutrients in the nasal passages. The death of nasal tissue cells can cause damage to the nose of drug users, resulting in changes in the facial area [41]. Therefore, our model captures this local feature. On the other hand, the facial features of PDUD in the cheek areas were directly related to the rapid loss of facial fat caused by illicit drug use, which is consistent with previous research [11,42]. The inhibitory effect of drugs on human appetite can lead to malnutrition, and the distribution of superficial fat on the face is mainly on the medial cheek fat and middle cheek fat [9]. Therefore, this feature of the change in facial fat distribution caused by drug use was also extracted and recognized by our model. The discovery of these facial features provides ideas for basic medical research, including mechanisms of drug action, facial anatomy characteristics, and physiology mechanism of PDUD.

There are several limitations to this approach. Limited by our research data, our study did not meticulously categorize PDUD in terms of the two attributes of illicit drug type and the time of suffering from drug use disorders. Moreover, the image information collected through the mHealth app would be affected by the hardware of mobile devices. The stability of the mHealth app will be optimized in further study. Nevertheless, our work is highly innovative in related fields with high feasibility and accessibility, especially in detecting and analyzing facial features in PDUD.

## 5. Conclusions

Drug use disorders continue to attract attention—however, there is a lack of simple and efficient tools in clinical screening, especially in primary care clinics. This paper is, to the best of our knowledge, the first study to apply the CNN model using transfer learning to screen PDUD by using facial images. Large-scale datasets were prepared, and various experiments were designs to optimize this model. The performance of this model was evaluated in real scenarios, and the results maintained high accuracy, sensitivity, and specificity. Therefore, this study is promising for clinical practice, which would help clinicians find potential PDUD and provide timely intervention and target treatment. This is also the first study to quantitatively analyze the facial features of PDUD. This contributes to the exploration of facial anatomy characteristics and physiological mechanisms of PDUD.

## Figures and Tables

**Table 1:** Experiments settings of CNN models with different parameters adjusted strategy.

Experiment	Backbone	Learning Rate	Batch Normalization	Pre-trained	Weight Initialization
Exp ^a^-1	Vgg-19	0.1	○	○	○
Exp ^a^-2	Vgg-19	0.01	○	○	○
Exp ^a^-3	Vgg-19	0.01	●	○	○
Exp ^a^-4	Resnet-18	0.1	●	○	○
Exp ^a^-5	Resnet-18	0.01	●	○	○
Exp ^a^-6	Resnet-18	0.1	●	○	●
Exp ^a^-7	Inception	0.01	●	○	○
Exp ^a^-8	Inception	0.1	●	○	○
Exp ^a^-9	Resnet-18 + Attention	0.1	●	○	○
Exp ^a^-10 *	Resnet-18	0.1	●	●	○

^a^—experiment, ●—with parameter, ○—without parameter, *—best experiment.

**Table 2:** Results of CNN models with freezing layers on the test dataset.

CNN Model	From Scratch (%)	Only Fully Connected (%)	The Last Five Convolutional Layers (%)
VGG-19	51.03	58.53	51.02
Inception	52.13	53.84	59.90
Resnet-18	60.43	60.97	74.63

**Table 3:** Results of CNN model on test dataset and external validation datasets.

Dataset	TP ^a^, n (%)	FN ^b^, n (%)	FP ^c^, n (%)	TN ^d^, n (%)	ACC ^e^ (%)	SEN ^f^ (%)	SPE ^g^ (%)
Test dataset	2637 (29.86)	362 (4.10)	991 (11.22)	4842 (54.82)	84.68	87.93	83.01
Validation 1 dataset	991 (27.53)	209 (5.81)	378 (10.50)	2022 (56.17)	83.69	82.58	84.25
Validation 2 dataset	11 (0.15)	2 (0.02)	749 (10.37)	6460 (89.45)	89.60	84.62	89.61
Validation 3 dataset	16 (0.15)	4 (0.04)	1136 (10.49)	9678 (89.33)	89.48	80.00	89.50
Validation 4 dataset	22 (0.15)	4 (0.03)	1456 (10.08)	12,962 (89.74)	89.89	84.62	89.90
Validation 5 dataset	27 (0.15)	5 (0.02)	1805 (10.00)	16,218 (89.82)	89.98	81.82	89.99
Validation 6 dataset	32 (0.15)	7 (0.03)	2156 (10.00)	19,471 (89.87)	90.02	82.05	90.03
Validation 7 dataset	39 (0.10)	6 (0.02)	3567 (9.88)	32,478 (90.00)	90.10	86.67	90.10

^a^ TP—true positive, ^b^ FN—false negative, ^c^ FP—false positive, ^d^ TN—true negative, ^e^ ACC—accuracy, ^f^ SEN—sensitivity, ^g^ SPE—specificity.

**Table 4:** Comparison of facial features in people with drug use disorders and the general population.

Facial Area	People with Drug Use Disorders		General Population	
	Number	Proportion (%)	Number	Proportion (%)
Left-eye	661	22.04 *	2095	35.92
Right-eye	786	26.21 *	2526	43.31
Mouth	936	31.21 *	2390	40.97
Nose	1289	42.98 **	1693	29.02
Left-cheek	1347	44.91 **	2004	34.36
Right-cheek	1345	44.85 **	2069	35.47

Note. * refers to the difference between the people with drug use disorders and the general population was significant (*p* < 0.001), and there is a higher proportion of the general population; ** refers to the difference between the people with drug use disorders and the general population was significant (*p* < 0.001), and there is a higher proportion of the people with drug use disorders.

**Figure A3:**
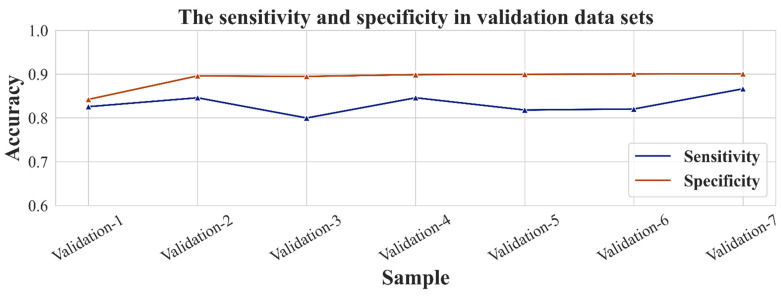
The results of sensitivity and specificity of the seven external validation datasets.

**Figure A5:**
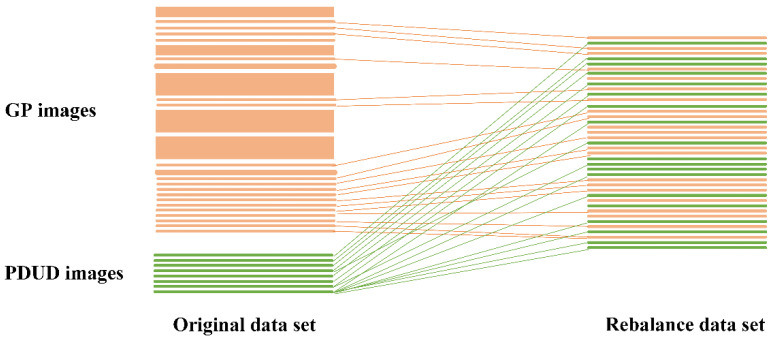
Resampling technique to rebalance the two-class distributions of images.

**Figure A6:**
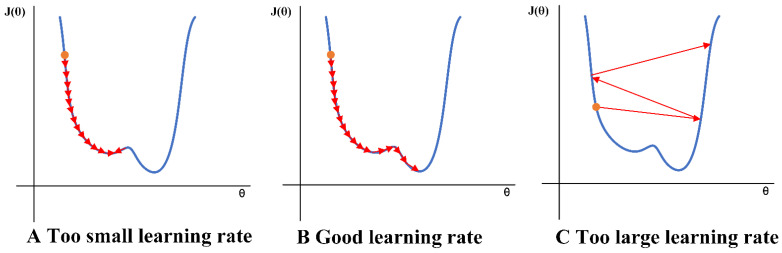
The influence of different learning rates on CNN model training.

**Figure A7:**
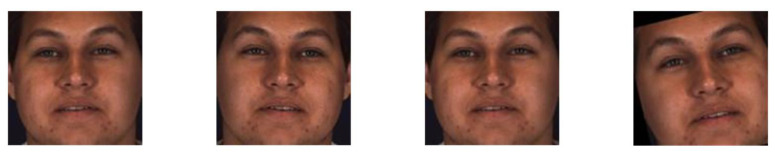
The image augmentation operation on the test dataset.

## Data Availability

The datasets during this study are available from the corresponding author on reasonable request.

## Abbreviations

PDUD	people with drug use disorders
GP	the general population
CNN	convolutional neural networks
DALYs	disability-adjusted life-years
EDs	emergency departments
DR	diabetic retinopathy
AUC	area under the curve
mHealth	mobile health
LR	learning rates
BN	batch normalization
SGD	stochastic gradient descent
Adam	adaptive moment estimation
Grad-CAM	gradient weighted class activation mapping

## Appendix A

### Appendix A.1. The Detailed Information of the App

The app was developed for PDUD and professional social workers of community drug treatment agencies by our team. The app’s registered users were PDUD who were from Qingyang District, Chengdu City, Sichuan Province, China. The social workers recruited PDUD who met the following criteria: (1) Currently undergoing community detoxification or community rehabilitation treatment; (2) had no severe physical disabilities or mental diseases; (3) had no difficulty in using smartphones; (4) willing to use the functions of our mHelath app and complete the informed consent document. During the study period, they voluntarily used this app and regularly uploaded their photos and videos data to record their daily lives, and these data could assist social workers in better assess the health status of PDUD. To facilitate social workers to better assess the status of PDUD, PDUD were encouraged to upload the images without makeup. This app was only used to collect data, and the CNN model did not run on the app in this study.

## Appendix B

### Appendix B.1. Model Architecture Analysis and Selection

The Vgg is a classical CNN architecture that utilizes small 3 × 3 filters to replace large ones. This helps it to decrease the number of parameters while keeping performance. The Vgg-19 consists of 16 convolutional layers with three fully connected layers and the same five pooling layers. The Inception replaced the 5 × 5 convolution with two 3 × 3 convolutions to decrease computational time and increase speed. The Resnet provided skip connections to solve the problem of the vanishing/exploding gradient. The Resnet group 2–3 convolutional layers as a single block called Resnet block and skip connection skipped training from a few Resnet blocks and connect directly to the output. Each Resnet block is two layers in the Resnet-18, and there are four groups Resnet blocks in the Resnet-18. In the study, the three architectures were be used as the backbone in transfer learning. The mini-batch size was set to 32, and used the mini-batch gradient descent method to calculate model error and update the model’s parameters. Due to the imbalance of samples in the training dataset and test dataset, the resampling technique was used to rebalance the class distributions of images in the two categories to avoid the model over-fitting (Figure A5) [43].

### Appendix B.2. The Adjusted Strategy of Model Training

Because the CNN models used stochastic gradient descent for optimization algorithm training, the LR was an important parameter that controls how much to change the model in response to the estimated error each time the weights are updated. LR that are too small may cause a long training process and become stuck, whereas a value too large may result in skipping the ideal minimum or an unstable training process (Figure A6). We chose 0.10 and 0.01 as the initial value for the LR and compared the training speed and accuracy on the dataset. Moreover, during the model training process, the parameter LR was dynamically adjusted. The LR decayed with a multiplicative factor of 0.10 was applied after every ten epochs. Therefore, the model’s parameters were updated with a larger learning rate in the early stage of training, and a lower learning rate in the later stage can help converge the optimization process. BN was designed to automatically standardize the inputs to a layer in a deep learning neural network for each mini-batch. BN had the effect of stabilizing the learning process and accelerating the model’s training process, and it can improve the performance of the CNN model. The accuracy of the three models with/without BN on the dataset was compared. The aim of weight initialization was to prevent layer activation outputs from exploding or vanishing during a forward pass through a deep neural network. At the beginning of training the model, the weights of each layer in the network were initialized with random numbers, which caused the loss gradient to be too large or too small during the training process, which meant that the accuracy of the model was difficult to keep improving as the training time increases. The Kaiming Initialization was chosen as the model initialization method. Compared with the random initialization weight in the layer, the Kaiming Initialization method ensured that the loss of the model continues to decrease during the training process. Finally, the cross-entropy function was configured as the loss function for models.

### Appendix B.3. The Optimization Algorithms Analysis

In the training process, the Adam and SGD (with momentum 0.90) algorithms were chosen as the optimizer to reduce the loss and accelerate the convergence speed of three CNN models. The accuracy of models with different optimizers on the test dataset was also compared.

### Appendix B.4. The Data Augmentation of Images

During the training process, three data augmentation techniques were applied to the images to improve model robustness and avoid over-fitting the model, which included image color jitter, image flipping, and image standardization. The brightness, contrast, and saturation of the images in the dataset were randomly changed. Then, the images randomly with 0.50 probability were horizontally flipped. Finally, a random affined transformation was applied to the images. (Figure A7).

In order to better understand the entire training process, the pseudocode was provided in Algorithm A1.

Accuracy is the number of correctly predicted data by the model out of all the data.

$$Accuracy=\frac{TP+TN}{TP+FP+FN+TN}.$$

Sensitivity refers to the proportion of actual PDUD that are correctly detected by the model, reflecting the model’s ability to detect patients with drug use disorders.

$$\mathrm{Sensitivity}=\frac{TP}{TP+FN}$$

Specificity refers to the proportion of actual general individuals that are correctly detected by the model, which reflects the ability of the model to exclude non-PDUD.

$$Specificity=\frac{TN}{FP+TN}$$

**Algorithm A1.** The pseudocodes of model training.
Input: load CNN model name MODEL, mini-batch size N, pre-trained config CONFIG, optimizer function OPTIM, loss function LOSS, data path PATH, training iteration EPOCH
1 model = LoadModel(MODEL)
2 **if** CONFIG has pre-trained parameters **then**
3 Load pre-trained parameters
4 **if** CONFIG need freeze some layers **then**
5 Set requires_grad = False
6 model.to(‘cuda:0’)
7 **if** CONFIG has optimizer parameters **then**
8 Set parameters of optimizer (include Adam or SGD)
9 optimizer = OPTIM(learning_rate=0.1/0.01)
10 **if** CONFIG need adjust learning rate **then**
11 Set lr_scheduler to adjust learning rate
12 **if** CONFIG has more fine-tuning setting **then**
13 Add other fine-tuning setting (e.g. Batch Normalization)
14 criterion = LOSS()
15 **if** PATH is valid **then**
16 Prepare train data loader
17 train_loader = DataLoader(batch_size=N)
18 Prepare validation data loader
19 valid_loader = DataLoader(batch_size=N)
20 **for** an epoch in EPOCH **do**
21 **for** traing data in train_loader **do**
22 train batch-size training data
23 zero gradients buffers
24 calculate training loss
25 backpropagate the error
26 update weight
27 **if** log training history **then**
28 Log accuracy and loss of each epoch in history
29 Test the trained model in validation data set
30 model.eval()
31 **for** validation data in valid_loader **do**
32 calculate the best accuracy
33 Save the trained model
34 Save procedure history

Resampling data was obtained by only examining some of the samples from a large number of images from one category, in order to balance the number of images of the two categories.

Learning rates that are too small can cause the training process to be too slow, making it difficult to reach the minimum point of the loss function. Whereas, learning rates that are too large can result in the minimum point of loss function being skipped, making the training process unstable.

**Table A1:** Results of 10 experiments of CNN model on the test dataset.

Experiments	The Best Accuracy of the Experiment (%)
Experiment-1	51.03
Experiment-2	51.25
Experiment-3	61.23
Experiment-4	79.72
Experiment-5	81.64
Experiment-6	60.43
Experiment-7	79.13
Experiment-8	50.55
Experiment-9	78.88
Experiment-10	84.68

**Table A2:** Results of the model with different optimization algorithms on the test dataset.

CNN Model	The Best Accuracy of the Experiment (%)
VGG-19 + Adam	51.02
VGG-19 + SGD	61.23
Inception + Adam	54.99
Inception + SGD	79.13
Resnet-18 + Adam	71.61
Resnet-18 + SGD	79.72

## Appendix C

### Appendix C.1. Convolutional Neural Network

A convolutional neural network (CNN) is an implementation of a neural network for machine learning that specializes in processing large-scale data, such as images, which is widely used in medical images applications. The typical CNN usually consists of input, feature extraction and classification, and output. Among them, feature extraction is the core component. It includes the convolutional layers, pooling layers, and non-linear activation units. Each convolutional layer contains various filters called kernels. The filter is a matrix of integers that are used on a subset of the input pixel values, the same size as the kernel. Each pixel is multiplied by the corresponding value in the kernel, and a single value is added to the result to simply represent the grid unit (such as a pixel) in the output channel/feature map [44].
